# Acceptability and factors associated with post-partum IUCD use among women who gave birth at bale zone health facilities, Southeast-Ethiopia

**DOI:** 10.1186/s40834-018-0071-z

**Published:** 2018-11-06

**Authors:** Alemayehu Gonie, Chanyalew Worku, Tesfaye Assefa, Daniel Bogale, Alemu Girma

**Affiliations:** 1 College of Health Sciences, Debre Berhan University, Debre Berhan, Ethiopia; 2 College of Medicine and Health Sciences, University of Gondar, Gondar, Ethiopia; 3 School of Health Sciences, Goba Referral Hospital, Madda Walabu University, Bale-Goba, Ethiopia; 4 School of Medicine, Goba Referral Hospital, Madda Walabu University, Bale-Goba, Ethiopia

**Keywords:** Acceptability, Intrauterine contraceptive device, Postpartum family planning

## Abstract

**Background:**

The postpartum intrauterine contraceptive devices (PPIUCD) is the only family planning method for couples requesting highly effective, reliable, inexpensive, non-hormonal, immediately reversible, and long-acting contraceptive that can be initiated during the immediate postpartum period and it has no a negative effect on lactation. Despite these benefit, the acceptance and utilization of immediate PPIUCD were very low and the reasons for rejecting immediate PPIUCD usage have not been characterized in Southeast Ethiopia. Therefore, this study determined the level of acceptability and factors associated with immediate PPIUCD use among women who gave birth at Bale zone health facilities, Southeast Ethiopia.

**Methods:**

A facility based cross-sectional study was conducted from March to July 2017 in Bale zone health facilities. Four hundred twenty-nine women were successfully interviewed using structured and pre-tested questionnaire. Health facilities were selected by lottery method. Study participants were selected systematically. Data were entered into Epi data version 3.1 and exported into SPSS version 21 for analysis. Logistic regression analyses were done. A significant association was declared at a *p*-value less than 0.05.

**Results:**

The acceptance of immediate PPIUCD usage was 12.4%. Non-acceptors reported their reasons for rejecting PPIUCD use; concern and fears of complications (24.8%), religious beliefs (19.8%), and husband refusal (17.7%). Respondents who had completed secondary education were more likely to accept PPIUCD usage than those who had no formal education (AOR = 3, CI = 11.81, 53.91). In addition, the odds of accepting PPIUCD insertion was higher among women who attended 3 antenatal care visits than those who did not attend antenatal care visits for the current birth (AOR = 1.81, CI = 0.34, 0.85).

**Conclusions:**

The acceptance of immediate PPIUCD usage was still low. This might be attributed to the low achievement of education, perceived concern and fears of complications towards IUCD insertion. The male partner’s refusal and religious beliefs also have a role in the usage of postpartum IUCD. Due attention should be given to enhancing educational level of women and effective IUCDs counseling should be given during antenatal care visits to correct misconceptions and fears of complication about PPIUCD insertion.

**Electronic supplementary material:**

The online version of this article (10.1186/s40834-018-0071-z) contains supplementary material, which is available to authorized users.

## Background

Postpartum family planning is a prevention of unintended and closely spaced pregnancies in the first 12 months after delivery. During the postpartum period, there is a high chance of having unplanned pregnancy which has an adverse outcome like abortion, premature labor, postpartum hemorrhage, low birth weight baby, fetal loss and maternal death [1, 2].

Postpartum intrauterine contraceptive device (PPIUCD) is the only family planning method which is highly effective, reliable, inexpensive, non-hormonal, immediately reversible, and long-acting contraceptive that can be initiated during the immediate postpartum period and it has no a negative effect on lactation [1–3]. PPIUCD can promote the health of the women and children by preventing financial, psychological, obstetric, and other health-related complications associated with closely spaced pregnancies [1]. Immediate PPIUCD insertion does not require repeated health care visits for contraceptive refills [4]. The insertion of immediate PPIUCD is easy and safe when compared with delayed postpartum and interval insertion of the intrauterine contraceptive device (IUCD) [5] and it can be initiated by a mid-level skilled birth attendant [6].

Women are highly motivated to accept family planning methods during the postpartum period [7]. The immediate postpartum period is a great opportunity for PPIUCD service providers to introduce the method especially in settings where women have cultural and/or geographical limitation to access contraceptive service [8]. Failure to provide immediate postpartum contraception can contribute the occurrence of unintended pregnancies because most of the women often do not return for postnatal services [9]. The initiation and provision of contraceptive methods during the immediate postpartum period safeguard the women from unintended pregnancy before they resume sexual activity or return to fecundity [10].

Despite these benefits of immediate PPIUCD, the acceptance and utilization of immediate PPIUCD were still very low in developing countries including Ethiopia. In Africa, only 4.6% of women utilized IUCD which was lower than the global utilization (13.9%) [11]. In Ethiopia, the provision of IUCD was free of costs, but its utilization was still very low in which only 2% of women utilized IUCD nationally [12]. Low acceptability of immediate PPIUCD usage could have an influence on high unmet need and low utilization of the contraception [13]. It was also known that the acceptability of contraceptive methods was fundamental to correct and consistent utilization [14]. Thus, acceptance determines the continued utilization of the chosen contraceptive method.

Numerous factors could contribute to low acceptance and utilization of immediate PPIUCD. Findings from other studies showed that lack of knowledge about the method, lack of trained providers and preference of short-acting contraceptive methods, spousal opposing, and fears of complication were the main reasons for not accepting PPIUCD use [15, 16]. A study in Adesh tertiary hospital, India woman’s and husband’s education, attending antenatal and postnatal visits were significantly associated with IUCDs utilization. Lack of knowledge and access to IUCD services, and fear of side effects were the reasons for not accepting of contraceptives during postpartum period [17]. In Telangana tertiary care hospital, acceptance of PPIUCD usage was higher among women who completed secondary education [18]. In New Delhi, the most common reason affecting acceptance of PPIUCD insertion was lack of husband involvement in family planning counseling services [19]. In Egypt, future pregnancy desire, preference of another contraceptive method, and fear of complications were the most common reasons for refusing PPIUCD use [20]. In Mekele city, fears of side effects and future fear of infertility were the main reasons for not accepting long-acting reversible contraceptive [14].

Even though various surveys have been done on the utilization and determinant factors of family planning, the reasons for not accepting immediate PPIUCD use have not been well characterized in southeast Ethiopia. Determining the level of acceptance measures the access to information, education and communication activities that have been done in the study settings. Similarly, information on the acceptability of immediate PPIUCD use may help identify program areas that need to be strengthened. Therefore, this study determined the level of acceptability and identified factors associated with immediate PPIUCD use among women who gave birth at Bale zone health facilities, southeast Ethiopia.

## Methods

### Study area and period

The study was conducted from March to July 2017 in 17 health facilities in Bale zone, Southeast Ethiopia. Robe, Bale zone city, is located 435-km away from Addis Ababa. Based on Bale zone health office report, the zone has 715 health facilities (1 referral hospital, 3 zonal hospitals, 84 health centers, 354 functional health posts, 179 private clinics, 4 public clinics). Family planning, antenatal care (ANC) and delivery services are provided free of charge in all public health facilities. Modern contraceptive methods (injectable, pills, implants, male condoms) are available in all health facilities. Currently copper T 380A IUCD is available in health centers and hospitals, and permanent contraceptive is available in hospitals (*unpublished Bale zone health office report 2017*).

### The study design

A facility based cross-sectional study design was employed.

### Study population

Postnatal women who gave birth at Bale zone health facilities during the study period were considered as the study population. Postnatal women who did not fulfill World Health Organization medical eligibility criteria for IUCD insertion were excluded.

### Sample size and sampling procedure

The sample size was determined using single population proportion formula with the following assumptions: proportion of women who accepted long-acting contraceptive as 16.4% (*p* = 0.164) taken from a study conducted in Mekele city [14], with 95% confidence interval (CI) to be 1.96, and margin of error to be 5%. Adding non-response rate of 10% and considering the design effect of 2, a total sample size was 465 women.

Twenty percent of the health facilities were selected based on the proposed sample fraction guideline for assessing the operation of district health system [21]. Based on this guideline, 13 health centers and 4 hospitals were selected by lottery method. The sample size for each health facility was determined by proportionate allocation. Study participants were selected systematically. The sampling interval was determined by dividing the number of average monthly delivery services by its sample size. The first woman was selected by lottery method from their order of discharge registration, and every third woman at the exit of the health facility was included in the study.

### Data collection

The structured and pre-tested questionnaire was prepared first in English from peer-reviewed articles and then translated into Amharic and Afan Oromo (local languages). The questionnaires comprise four sections; maternal socio-demographic factors, obstetric characteristics, knowledge, and attitude of women towards PPIUCD usage Additional file 1. Data collectors were trained for 2 days before the actual data collection. The training was focused on PPIUCD counseling, understanding of the questionere, ethical conduct, and identifying of eligible women.

### Operational definitions

- Acceptance of IUCD: woman’s verbal consent to use IUCD within 10 min to 48 h of delivery of placenta after they counseled about PPIUCD [9].
- Knowledge: the fact that respondents know about PPIUCD as a method of birth spacing and its benefits. It was measured by calculating the mean score of 10 items and categorized as having a good knowledge (if the participant answered greater than the mean score of knowledgeable questions) or not having a good knowledge (if the participant scored less than mean score of knowledgeable questions).
- Attitude: the ways that respondents think and behave about PPIUCD use. It was measured by calculating the mean score of 6 items and categorized as having a good attitude (if the participant answered greater than mean score of attitudinal questions) or not having a good attitude (if the participant scored less than mean score of attitudinal questions).

### Data processing and analysis

Data were checked for completeness and inconsistencies. Epi-data version 3.1 was used for data entry and data were exported into SPSS version 21. Descriptive statistics were computed. Bi-variable and multivariable logistic regression analyses were done to identify the relationship between dependent and independent variables. Independent variables that had a significant association in the bivariable analysis were entered into the multivariable analysis. In the final model, a significant association was declared at a *p* < 0.05. The results were presented in text, graph, and tables with adjusted odds ratio (AOR) and the corresponding 95% confidence interval.

### Ethical considerations

Ethical approval was obtained from a research review committee of Madda Walabu University. Letters were secured from Bale zone Health Bureau and respective health facilities. Written informed consent was obtained from each study participant. All information obtained from each study participant was kept confidential throughout the process of study, and the name of the participant was replaced by code. Withdrawal from the study at any point if they wished was assured.

## Results

### Maternal socio-demographic characteristics

A total of 429 women were successfully interviewed which made a response rate of 92.3%. About 30% of women were in the age group of 21–25 years. The mean age of study participants was 26.26 (±4.78 SD). More than three-fourths (76.2%) of the study participants were Muslim and 19.3% were Orthodox Christians. The largest proportions, 93.9% of women were married. Nearly half of study participants (47.8%) had no formal education while (22.1%) had completed primary education. Regarding the occupation of women and their husbands, almost equal proportions (56.9% of women versus 60.2% of husbands) were housewives and farmers, respectively. Fifty-seven percent of women were living in urban areas (Table 1).

**Table 1:** Socio-demographic distributions of women who gave birth at Bale zone health facilities, Southeast Ethiopia, August 2017

Characteristics	Categories	Number	Percent
Age of respondents (in years)	≤ 20	76	17.7
	21–25	127	29.6
	26–30	123	28.6
	31–35	80	18.7
	≥36	23	5.4
Marital status	Married	403	93.9
	Single/separated	26	6.1
Religion	Muslim	327	76.2
	Orthodox	83	19.3
	Protestant	19	4.5
Educational level of women	No Formal Education	199	46.4
	Primary Education	102	23.8
	Secondary Education	67	15.6
	College Education	61	14.2
Educational level of husband	No Formal Education	196	45.7
	Primary Education	114	26.6
	Secondary Education	58	13.5
	College Education	61	14.2
Occupation of women	Housewife	244	56.9
	Farmer	62	14.5
	Merchant	66	15.4
	Employed	57	13.3
Occupation of husband	Farmer	258	60.2
	Employed	82	19.2
	Merchant	78	18.1
	Others^b^	11	2.5
Residence	Urban	246	57.4
	Rural	183	42.6

^b**=**^daily laborers, drivers

### Obstetrics characteristics of respondents

About 79.2% of women were multigravida (had more than one pregnancy) and 73.6% were multiparous. On average, women had three (±2.24 SD) live children. The mean number of future pregnancy desire among women was 3.8 (±1.79 SD). Thirty-nine percent of women had attended 4 ANC visits while 12.4% of them had not attended ANC visits for the current birth. Around 53% of study participants used contraceptives before the current birth (Table 2).

**Table 2:** Obstetrics characteristics of women who gave birth at Bale zone health facilities, Southeast Ethiopia, August 2017

Characteristics	Categories	Number	Percent
Gravida	Primigravida	68	20.8
	Multigravida	259	79.2
Parity	Primipara	113	26.4
	Multipara	316	73.6
Antenatal care visits	Didn’t attend ANC visits	54	12.6
	One	38	8.4
	Two	75	17.5
	Three	94	21.9
	Four	170	39.6
Status of birth	Planned	376	87.6
	Unplanned	53	12.4
Average number of live birth	3.4 (±2.24 SD)		
Average future pregnancy desire	3.8 (±1.79 SD)		
Contraceptives use before current birth	Yes	231	53.6
	No	198	46.2
Which type (*n* = 231)	Pill	24	10.4
	Implant	152	65.8
	Injectable	51	22.1
	IUCD	4	1.7
Who decided the use of modern FP (*n* = 231)	Wife	38	16.5
	Husband	18	7.8
	Both of us	175	75.8

### Knowledge and attitude of women towards PPIUCD usage

The majority of study participants (76.4%) have ever heard about IUCD as a contraceptive method and 63.2% of them responded that they have ever heard IUCD can be inserted immediately after delivery. Of women who have ever heard about IUCD as a contraceptive method, half of them correctly answered the duration of pregnancy protection. IUCD could be put in the uterine (80.7%), has no risk of getting sexually transmitted infections (40.8%) and has no interference with sexual intercourse (43.2%) were highly rated. The mean score of correctly answered knowledge questions was (13.74 ± 2.8 SD). Fifty-four percent of participants (54.5%) scored above the mean score and they were considered as having good knowledge while 45.5% scored below the mean score and were considered as not having good knowledge (Table 3).

**Table 3:** Knowledge of PPIUCD insertion among women who gave birth at Bale zone health facilities, Southeast Ethiopia, August 2017

Variables	Category	Number	Percent
Ever heard about IUCD as a contraceptive method	Yes	327	76.3
	No	102	23.7
Ever heard IUCD can be inserted immediately after delivery? (*n* = 327)	Yes	206	63.0
	No	121	37.0
IUCD prevents unwanted pregnancy for at least 3 years? (*n* = 327)	Yes	134	40.9
	No	58	17.7
	Don’t know	135	41.4
IUCD is FP method that can be put into uterine (*n* = 327)	Yes	264	80.7
	No	14	4.3
	Don’t know	49	15.0
IUCD has no high risk of getting sexually transmitted infections (*n* = 327)	Yes	133	40.8
	No	71	21.6
	Don’t know	123	37.6
IUCD has no interference with sexual intercourse (*n* = 327)	Yes	141	43.1
	No	83	25.4
	Don’t know	103	31.5
IUCD is immediately reversible (*n* = 327)	Yes	128	39.2
	No	102	31.2
	Don’t know	97	29.6
IUCD cannot cause cervical cancer (*n* = 327)	Yes	124	38.0
	No	57	17.4
	Don’t know	146	44.6
Overall PPIUCD knowledge level	Having a good knowledge	178	54.4
	Not having a good knowledge	149	45.6

Regarding the attitude of participants towards the use of PPIUCD, they agreed that insertion & removal of IUCD is highly painful (33.6%), insertion of IUCD causes loss of privacy (33.6%), and using IUCD restricts normal activities (27.4%). The mean score of correctly answered attitudinal questions were (8.74 ± 2.46). Twenty-eight percent of participants (28.9%) scored above the mean score and they were considered as having a good attitude while 71.1% scored below the mean score and were considered as not having a good attitude (Table 4).

**Table 4:** The attitude of PPIUCD insertion among women who gave birth at Bale zone health facilities, Southeast Ethiopia, August 2017 (*n* = 327)

Variables	Category	Number	Percent
Do you think insertion & removal of IUCD is highly painful?	Agree	109	33.4
	Disagree	71	21.7
	Don’t know	147	44.9
Using IUCD cause irregular bleeding	Agree	69	21.1
	Disagree	86	26.3
	Don’t know	172	52.6
Do you think the insertion of IUCD cause to lose privacy	Agree	123	37.6
	Disagree	106	32.4
	Don’t know	98	30.0
Using IUCD restrict normal activities	Agree	63	19.3
	Disagree	139	42.5
	Don’t know	125	38.2
IUCDs may impair future fertility	Agree	136	41.6
	Disagree	87	26.6
	Don’t know	104	31.8
Overall PPICD insertion attitude level	Having good attitude	95	28.9
	Having poor attitude	232	71.1

### Acceptance of PPIUCD insertion

**Fig. 1:**
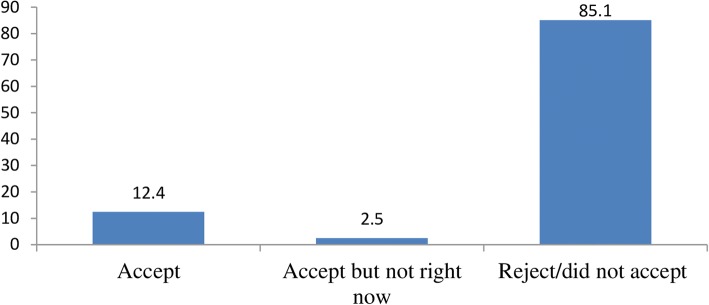
Acceptance level of immediate PPIUCD usage among women who gave birth at Bale zone health facilities, Southeast-Ethiopia, 2017

**Fig. 2:**
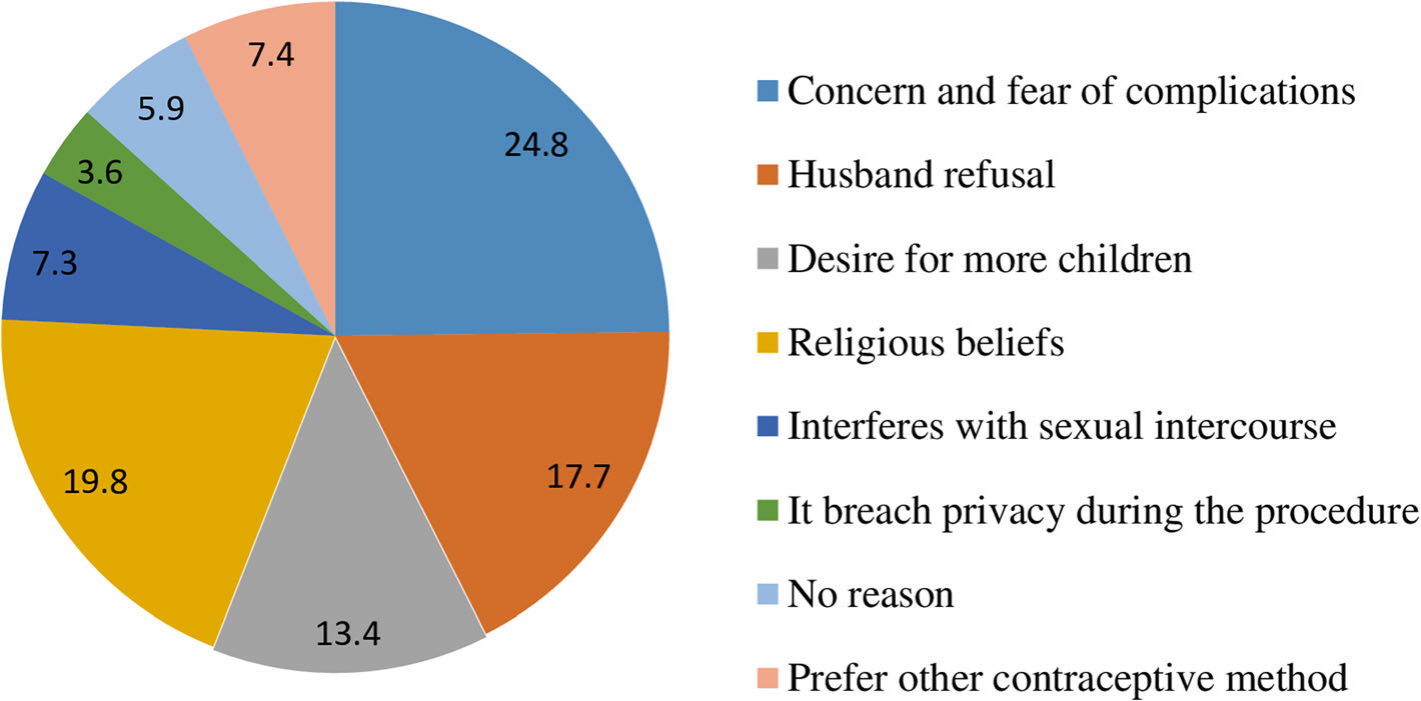
Reasons for rejecting immediate PPIUCD usage among women who gave birth at Bale zone health facilities, Southeast-Ethiopia, 2017

The overall acceptance of immediate PPIUCD usage was 12.4% (Fig. 1). Non-accepters reported their reasons for rejecting PPIUCD use; concern and fear of complications (24.8%), religious beliefs (19.8%), and husband refusal (17.7%) (Fig. 2).

### Factors associated with acceptance of PPIUCD usage

Ever heard IUCD can be inserted immediately after delivery, ANC visits, educational level, and age of the women showed significant association in the bivariable analysis. In the multivariable analysis, had been completing secondary education and attending 3 ANC visits were associated with the acceptance of PPIUCD insertion. It was observed that women who had completed secondary educational level were 3 times more likely to accept PPIUCD usage than women who had no formal education (AOR = 3, CI = 11.81, 53.91). In addition, the odds of accepting PPIUCD use was higher among women who attended 3 ANC visits than those who did not attend ANC visits before the current birth (AOR = 1.81, CI = 0.34, 0.85) (Table 5).

**Table 5:** Factors associated with the acceptance of PPIUCD use among women who gave birth at Bale zone health facilities, southeast Ethiopia, August 2017

Variable	Category	Acceptance of PPIUCD use		COR (95% CI)	AOR (95% CI)
		Accept	Reject		
Age of the women in years	≤20	7	69		1
	21–25	12	115	1.03(0.47, 3.95)	1.32(0.35, 14.73)
	26–30	22	101	2.15(0.27, 8.91)	1.94(0.17, 7.90)
	31–35	15	65	2.27(0.27, 0.91)	2.14(0.67, 9.42)
	≥36	4	19	2.07(0.27, 0.91)	1.88(0.71, 21.04)
Educational level of women	No Education	18	181		1
	Primary	14	88	1.60(3.14, 18.01)	1.20(0.51, 4.37)
	Secondary	16	51	3.15(1.02, 31.2)	3.10(11.81,53.91)*
	College	12	49	2.46(12.11, 35.2)	1.39(0.85, 5.07)
ANC follow up	No ANC visit	6	48		1
	One	10	28	2.86(0.40, 32.51)	2.73(0.31, 23.81)
	Two	13	62	1.68(2.14, 17.36)	1.80(0.21, 15.36)
	Three	16	78	1.64(0.14, 0.86)	1.81(0.34, 0.85)*
	Four	14	156	0.41 (0.38, 9.19)	0.17(0.31, 4.98)
Ever heard IUCD can be inserted immediately after delivery	Yes	44	162	0.56(7.10, 38.86)	1.59(0.59, 5.77)
	No	16	105		1

*P-value less than 0.05 in multivariable analysis, 1 = Reference

## Discussions

In this study, it was found that the overall acceptance of PPIUCD use in the study population was 12.4%. This was slightly comparable with findings in Central India (11.9%), tertiary care center, Indore (10.0%), but lower than other studies conducted in Zenana hospital, Jaipur (21.8%), Jorhat tertiary care hospital, Assam (36.6%), Faridabad district, India (39.0%) and Cuttack medical college, Odisha (25.32%) [22–26]. It was also higher than Jay Kay Lon hospital, Kota (2.94%) [16]. This variation of acceptance rate might be due to the difference in the level of awareness, educational level of respondents, religious beliefs and various misconceptions about PPIUCD insertion in the study settings.

In our study, the most common perceived reasons of rejecting immediate PPIUCD use reported by the respondents were concern and fear of complications (24.8%), religious beliefs (19.8%), and husband refusal (17.7%). This finding was supported by other studies conducted at tertiary care hospital, Telangana [27]. A similar observation was reported by Kumari Saroj and Goyal Neha where fear of side effect and complication (32.5%) were the most common reason to reject PPIUCD usage [23]. A study conducted by Sharma A et al. revealed that fear of complication (69.96%) was the reason for refusal [24]. According to Priya et al., the belief that PPIUCD insertion might deter the future conception was the reason for refusal among multiparas’ (65.0%) [28]. This implies that the presence of overwhelming perception towards fear of complication, and religious unacceptability of PPIUCD use by women in the study settings.

In this study, the women who had attended secondary educational level were 3 times more likely to accept PPIUCD use than those women who had no formal education. This agreed with a study reported by Sangeetha Jairaj and Sridhar Dayyala where completing secondary education determined acceptance of IUCD use [18]. Maluchuru S et al. found that primary education affected acceptance of IUCD use [29]. This observation suggests that education has a positive influence on women’s interest to accept PPIUCD use including their FP utilization.

In our study, the odds of accepting PPIUCD use was higher among women attending 3 ANC visits after the delivery. A similar finding was reported by Kumari Saroj and Goyal Neha where antenatal care played a significant role in the acceptance of PPIUCD use [23]. Shashi Kant et al. reported that having attended ANC visits were more likely to accept PPIUCD use [24]. The possible reason why women who attended 3 ANC visits accepted PPIUCD was probably they might be counseled by healthcare workers during their ANC visits. It could be also explained that during ANC visits, health care providers clarified misconceptions about PPIUCD use. Therefore, providing effective contraceptive counseling during ANC visits could address any misperceptions and motivate the women for accepting PPIUCD use immediately after delivery.

## Conclusions

In this study, the acceptance of immediate PPIUCD usage was still low. This might be mainly attributed to the low achievement of education, perceived concern and fears of complications towards IUCD insertion. The male partner’s refusal and religious beliefs also have a significant role in the usage of postpartum IUCDs. Had been completing secondary education and attending 3 antenatal care visits before the current birth were associated with acceptance of immediate PPIUCD use. Therefore, due attention should be given to enhancing educational level of women and effective IUCD counseling should be given during the antenatal care visits to correct misconceptions and fears of complication about PPIUCD insertion. In addition, continuous education and awareness creation session should be arranged to correct perceived fears of PPIUCD complications at the community level. This study was conducted in health facilities; hence the findings might not adequately reflect the entire population. For those women who heard about PPIUCD only during the immediate postpartum period, it might be difficult to make an informed decision towards acceptance of PPIUCD usage.

## Additional file

Additional file 1: The English version of consent form and questionnaire. (DOCX 23 kb)

## Abbreviations

ANC: Antenatal Care
AOR: Adjusted Odds Ratio
CI: Confidence Interval
FP: Family Planning
IUCD: Intrauterine Contraceptive Device
PPIUCD: Postpartum Intrauterine Contraceptive Device
SD: Standard Deviation
