# Supplementary material for: Acceptability and factors associated with post-partum IUCD use among women who gave birth at bale zone health facilities, Southeast-Ethiopia
Source: Contracept Reprod Med. 2018 Nov 6;3:16. doi: 10.1186/s40834-018-0071-z (PMC6219260; doi:10.1186/s40834-018-0071-z)
Supplement: Supplementary file 1 — The English version of consent form and questionnaire. (DOCX 23 kb) [file 40834_2018_71_MOESM1_ESM.docx]

# **12. Annexes**

**12.1. Consent Form and Questionnaire**

Dear Madam -----------------------------

I am ------------ and currently working at Goba referral hospital, Madda Walabu university. The aim of this study is assessing acceptability and factors associated with post-partum IUCD use among mothers who will give birth at Bale zone Health Facilities, Ethiopia. Findings from this research help health care provider to prepare appropriate educational material in such a way that postnatal mothers will be motivated to accept IUCD. This study will also enable to advise policy makers on strategies to enhance positive factors (to establish programs that are dedicated in educating parturient and promote PPIUCD) and remove negative factors that influence PPIUCD use (i.e. to adopt policies that dispel the parturient misbelieves), so as to increase contraceptive prevalence and ensure an increase in the PPIUCD choice of methods.

Thus this interview is prepared for this purpose to get appropriate information on the topic. The information that will be obtained using this interview will be used only for research purpose. Confidentiality and anonymity is fully assured, as your name is not required and only the research team will have access to the results. It will not affect you in anyway, should you not take part in this study? If yes continue to sign consent form If No, stop here

I have been informed that the purpose of this study is assessing acceptability and factors associated with post-partum IUCD use among mothers who will give birth at Bale zone Health Facilities, Ethiopia. I have understood that participation in this study is entirely voluntarily. I have been told that my answer to the question will not be given to anyone else and no reports of this study identify me in any way. I understood that participation in this study does not involve risks. I understood that Alemayehu Gonie is a contact person if I have question about the study or about my right as a study participant.

Respondent’s Signature ___________Date___________Start interview.

Supervisor’s name ________________ signature _________

**Address of investigators:** **Tell**: 0912379531 **e-mail:** [alemayehugonie19@gmail.com](mailto:alemayehugonie19@gmail.com)

| Q. N^o^  --- | Code No፡ 1= Accept 2= Accept but not right now 3=Reject | Facility Name----------- |
| --- | --- | --- |

**Instruction to the interviewer:** circle the number in front of the option based on the response

| **Sr.** | **Part I: Demographic Characteristics** | | **Options** | | | **Remark** | | |
| --- | --- | --- | --- | --- | --- | --- | --- | --- |
| 1.1 | Age of respondents (in years) | | ------- | | |  | | |
| 1.2 | Marital status | | 2= Married 1=Single/separated | | |  | | |
| 1.3 | Religion | | 1= Muslim 2= orthodox 3= protestant | | |  | | |
| 1.4 | Educational status of woman | | 1= Illiterate 2=primary education 3=secondary education 4= college education | | |  | | |
| 1.5 | Educational status of husband | | 1= Illiterate 2= primary education 3=Secondary education 4= college education | | |  | | |
| 1.6 | Occupational status of women | | 1= Housewife 2= Farmer 3= Merchant  4=Employee (government/private employee) | | |  | | |
| 1.7 | Occupational status of husband | | 1= Farmer 2= Merchant  3=Employee (government/private employee) | | |  | | |
| 1.8 | Residence | | 1=Urban 2= Rural | | |  | | |
| **2.** | **Part II: Obstetrics Characteristics** | | | | |  | | |
| 2.1 | Gravida (in number) | | | ------------ | |  | | |
| 2.3 | Parity (in number) | | | ------------ | |  | | |
| 2.4 | How many ANC follow up did you have? | | | 1=1 2=2 3=3 4=4 5=no | |  | | |
| 2.5 | Status of birth | | | 1=wanted 2= unwanted | |  | | |
| 2.6 | Number of live children (in number) | | | ----------- | |  | | |
| 2.7 | Future pregnancy desire (in number) | | | -------- | |  | | |
| **3.** | **Part III: knowledge Assessment** | | |  | |  | | |
| 3.1 | Did you use contraceptives before this birth? | | | 1= yes 2= no **If no skip to Q 3.4** | | | | |
| 3.2 | If yes for Q 3.1 which type? | | | 1=Pill 2=Injectable 3=Implant 4= IUCD 5=condom | |  | | |
| 3.3 | Who decide use of FP | | | 1=Wife 2=Husband 3=Both of us | |  | | |
| 3.4 | Have you ever heard about IUCD? | | | 1= yes 2=no **If no skip to Q 3.7** | | | | |
| 3.5 | If yes for Q 3.3, from where did you heard? | | | 1=From media (radio, TV) 2=Relative/friend  3=From health facility (health worker) | | | |  |
| 3.6 | Have you ever heard IUCD can be inserted immediately after delivery? | | | 1= yes 2=no | | | |  |
| **Do you agree(Yes) or disagree(No) with the following statements about IUCDS (tick one)** | | | | | | | |  |
| 3.7 | | IUCD prevents unwanted pregnancy for at least 3 yrs | | | 1= yes(agree) 2=no(disagree) 3= don’t know | | |  |
| 3.8 | | IUCD is FP method that can be put into uterine | | | 1= yes(agree) 2=no(disagree) 3= don’t know | | |  |
| 3.9 | | IUCD has **no** high risk of getting STIs | | | 1= yes(agree) 2=no(disagree) 3= don’t know | | |  |
| 3.10 | | IUCD has **no** interference with sexual intercourse | | | 1= yes(agree) 2=no(disagree) 3= don’t know | | |  |
| 3.11 | | IUCD is immediately reversible | | | 1= yes(agree) 2=no(disagree) 3= don’t know | | |  |
| 3.12 | | IUCD cannot cause cancer | | | 1= yes(agree) 2=no(disagree) 3= don’t know | | |  |
| **Note** | | Ask her What misconception she has about IUCD? Please write it either in Amharic or Afaan oromo | | | | | | |
| **4** | | **Attitude assessment** *(Do you agree or disagree with the following statements about IUCDS (tick one)* | | | | | | |
| 4.1 | | Insertion& removal of IUCD is highly pain full | | 1= yes(agree) 2=no(disagree) 3= don’t know | | |  | |
| 4.2 | | Using IUCD cause irregular bleeding | | 1= yes(agree) 2=no(disagree) 3= don’t know | | |  | |
| 4.3 | | Insertion of IUCD cause to lose privacy | | 1= yes(agree) 2=no(disagree) 3= don’t know | | |  | |
| 4.4 | | Using IUCD restrict normal activities | | 1= yes(agree) 2=no(disagree) 3= don’t know | | |  | |
| 4.5 | | IUCDs may impair future fertility | | 1= yes(agree) 2=no(disagree) 3= don’t know | | |  | |
|  | | **Counseling about IUCD** | |  | | |  | |
| 5.1 | | Do you agree to use IUCD today? | | 1. Yes 2. No **If no skip to Q 5.3** | | | | |
| 5.1 | | Why you **prefer** IUCD from other FP methods?  (circle all possible answers)  **(*only for accepters)*** | | 1= IUCD is long acting/term  2=it is safe 3=IUCD needs few follow up  4= it is reversible 5=No interference with BF | | | |  |
| 5.2 | | What is your reasons for accepting IUCD  (circle all possible answers)  **(*only for accepters)*** | | 1=For child spacing  2=Prevention of future pregnancies  3=Doctors/nurses advice 4=Friends use it | | | |  |
| 5.3 | | What is your reasons for **rejecting** IUCD (circle all possible answers)  ***(only for none accepters)*** | | 1=Satisfied/preferred other FP method  2=fear of complications  3=Family refusal/Husband object it  4=Desire for more children  5= Religious beliefs  6=Interferes with sexual intercourse  7=due to it breach privacy 8=No reason  9= other, specify ----------- | | | |  |
